# Clinical Relevance of Resection Margins in Patients with Total Laryngectomy or Laryngopharyngectomy

**DOI:** 10.3390/cancers16112038

**Published:** 2024-05-28

**Authors:** Simone E. Bernard, Cornelia G. F. van Lanschot, Aniel Sewnaik, Maria A. J. de Ridder, Jose A. Hardillo, Dominiek A. Monserez, Robert J. Baatenburg de Jong, Senada Koljenović

**Affiliations:** 1 Department of Otorhinolaryngology and Head and Neck Surgery, Erasmus MC Cancer Institute, University Medical Center Rotterdam, P.O. Box 2040, 3000 CA Rotterdam, The Netherlands; 2 Department of Medical Informatics, Erasmus MC, University Medical Center Rotterdam, P.O. Box 2040, 3000 CA Rotterdam, The Netherlands; 3 Department of Pathology, Erasmus MC, University Medical Center Rotterdam, P.O. Box 2040, 3000 CA Rotterdam, The Netherlands; 4 Department of Pathology, Antwerp University Hospital, Drie Eikenstraat 655, B-2650 Edegem, Belgium; 5 Faculty of Medicine, University of Antwerp, Universiteitsplein 1, B-2610 Antwerpen, Belgium

**Keywords:** laryngeal cancer, hypopharyngeal cancer, resection margins, squamous cell carcinoma, Royal College of Pathologists

## Abstract

**Simple Summary:**

Achieving margins of >5 mm is challenging in the larynx and hypopharynx because resections are constrained by their complex anatomy. The aim of this study was to retrospectively assess the clinical relevance of resection margins defined by the RCP in total laryngectomies (TLs) and total laryngopharyngectomies (TLPs). Similar survival rates for close and clear margins for primary and recurrent LSCC were found. This may suggest that a margin > 5 mm is not clinically relevant in terms of survival, therefore a margin of 1–5 mm should be accepted in certain subsites. Margins < 1 mm are related to significantly worse outcomes and should be avoided.

**Abstract:**

Background: Laryngeal and hypopharyngeal cancer is complex and resection margins are therefore constrained. The aim of this study was to investigate the clinical relevance of resection margins in laryngeal and hypopharyngeal surgery. Methods: A retrospective cohort study was performed for patients treated with a total laryngectomy (TL) or laryngopharyngectomy (TLP) for laryngeal or hypopharyngeal squamous cell carcinoma (LSCC and HSCC, respectively). Within the groups primary LSCC, recurrent LSCC, primary HSCC, and recurrent HSCC the relationship between the status of the resection margin according to the Royal Collage of Pathology and the recurrence and survival rates were investigated. Results: Positive resection margins were found in 54% for primary LSCC, 29% for recurrent LSCC, 62% for primary HSCC, and 44% for recurrent HSCC. For primary and recurrent LSCC, there was a linear association between total recurrence and narrowing margins (*p* = 0.007 resp. *p* = 0.008). Multivariate survival analysis for primary and recurrent LSCC showed a significantly worse disease free and disease-specific survival in case of positive margins compared to clear margins. Conclusion: Similar survival rates were recorded for close and clear margins for primary and recurrent LSCC. This may suggest that a margin > 5 mm is not clinically relevant in terms of survival. Therefore, a margin of 1–5 mm should be accepted in certain subsites. Margins < 1 mm are related to significantly worse outcomes and should be avoided.

## 1. Introduction

Surgery is an important treatment modality for head and neck squamous cell carcinoma in addition to (chemo)radiation. The goal of surgery is to achieve adequate resection margins (i.e., the distance between the tumor border and resection surface), as this is a crucial prognostic factor [1,2]. For head and neck cancer, resection margins are defined by the Royal College of Pathologists (RCP) as follows: clear >5 mm, close 1–5 mm, and positive < 1 mm [3]. However, achieving margins of >5 mm is challenging in the larynx and hypopharynx because resections are constrained by their complex anatomy. A balance between achieving adequate margins for better outcomes in terms of recurrence and survival versus sparing healthy tissue to maintain function and aesthetics is essential.

There is limited evidence for the clinical relevance of resection margins, as defined by the RCP, in laryngeal and hypopharyngeal cancer. Moreover, the published studies did not show an association between clear margins (>5 mm) and overall survival (OS) (*p* = 0.286) [4] or disease-free survival (DFS) (*p* = 0.11) [5], nor did resection margins appear to be an independent predictor for disease-specific survival (DSS) or recurrence in univariate or multivariate analysis [5,6]. However, surgeons always strive to prevent a positive margin (<1 mm) because this impairs prognosis [7,8]. In an earlier study, we determined the resection surfaces and described the maximum feasible resection margins for the larynx and hypopharynx per tumor location. In that study, we reported that a >5 mm margin is not always feasible for all resection surfaces because of the anatomy and limited thickness of the different tissue layers. A margin of >1 mm should be accepted for specific tumor subsites in the larynx and hypopharynx [9].

The aim of the current study was to retrospectively assess the clinical relevance of resection margins defined by the RCP, in total laryngectomy (TL) and total laryngopharyngectomy (TLP).

## 2. Materials and Methods

Inclusion criteria. Based on the medical records, a retrospective cohort study was performed at the Erasmus MC Cancer Institute, Netherlands (EMC). Patients treated with TL or TLP for primary or recurrent squamous cell carcinoma between January 2008 and July 2017 were included. Patients were excluded if they had an additional simultaneous head and neck tumor. This study was approved by the Medical Ethics Committee (MEC-2017-336).

Patient and tumor characteristics. A database was created based on patient characteristics (e.g., age, and sex), tumor characteristics (i.e., location, c/pTNM histological characteristics including differentiation grade, infiltration pattern, perineural growth, and angio-invasion), primary treatment, resection margin status, outcome data on tumor recurrence (location and date), and the last date of follow-up or date of death. The last follow-up was defined as the last date on which the patient was confirmed alive and ended in February 2022. Follow-up time was measured from the date of treatment (i.e., surgery or (chemo)radiation) until the last follow-up. The resection margins were recorded from the final pathology report (in millimeters) with respect to all resection surfaces (cranial, ventral, lateral, dorsal, and caudal). Total recurrence (TR) was recorded as the sum of local recurrence (LR) (i.e., around the stoma, in the neopharynx/esophagus/base of the tongue), regional recurrence (i.e., neck lymph nodes), and/or distant metastasis. Resection margins were defined according to the RCP guidelines as follows: clear > 5 mm, close 1–5 mm, and positive < 1 mm [3].

Statistical analyses. Statistical analyses were performed using IBM SPSS Statistics, version 21.0 for Windows, version 25 (IBM Corp., Armonk, NY, USA). A significance level of 5% was considered to be statistically significant. The study population was divided into four groups: primary laryngeal squamous cell carcinoma (LSCC), recurrent LSCC, primary hypopharyngeal squamous cell carcinoma (HSCC), and recurrent HSCC. Patient and tumor characteristics were compared using Pearson’s chi-square test for categorical variables and ANOVA for age. Univariate statistical analyses were performed separately for each group. Linear-by-linear association tests were used to determine the relationship between the resection margins and (local) recurrence. Disease-specific survival (DSS) and disease-free survival (DFS) were analyzed using Kaplan–Meier estimates, and differences in survival with respect to margin status were tested using the log-rank test. DSS was defined as the percentage of patients who did not die from LSCC or HSCC. DFS was defined as the time (months) after treatment without (recurrent) disease. Multivariate Cox survival analysis was performed for LSCC (primary and recurrent) and HSCC (primary and recurrent). In these models, confounders (candidate confounders: age, primary/recurrent tumor, pT, pN, and postoperative adjuvant treatment) were selected using the mean squared error method. Next to this retrospective cohort study, we performed an extensive literature search in the Medline, Embase, and Cochrane Collaboration databases regarding the clinical relevance of resection margins in the larynx and hypopharynx in oncologic surgery.

## 3. Results

### 3.1. Results overall

**Figure 1:**
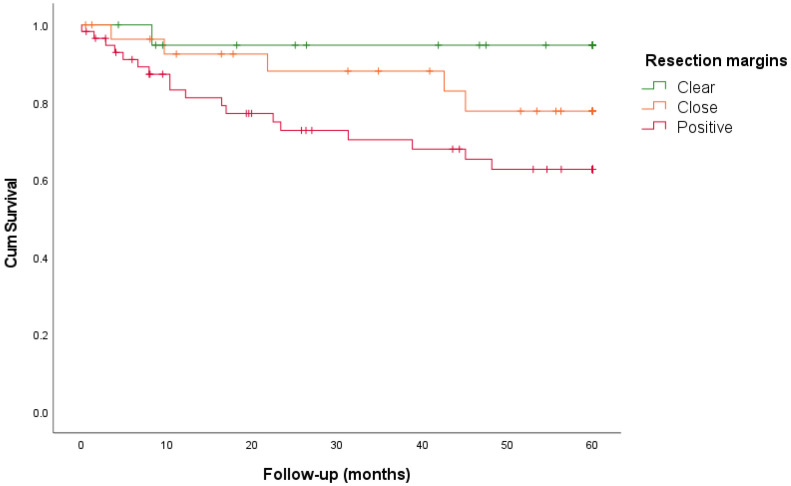
Relationship between disease-specific survival and resection margins for primary laryngeal squamous cell carcinoma.

**Figure 2:**
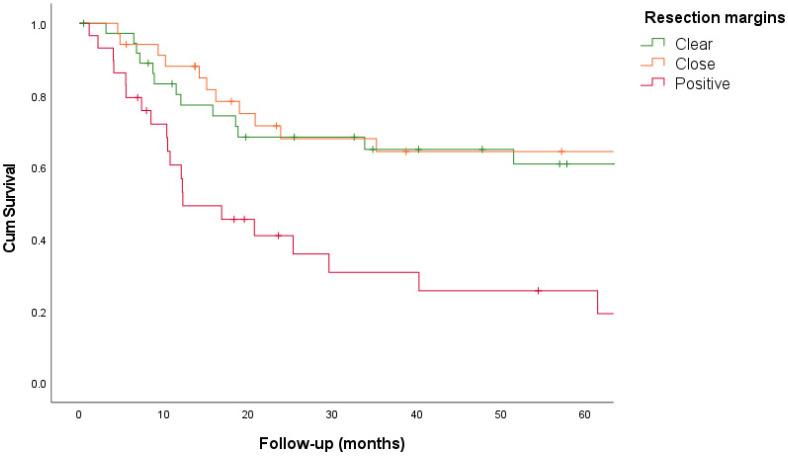
Relationship between disease-specific survival and resection margins for recurrent laryngeal squamous cell carcinoma.

**Figure 3:**
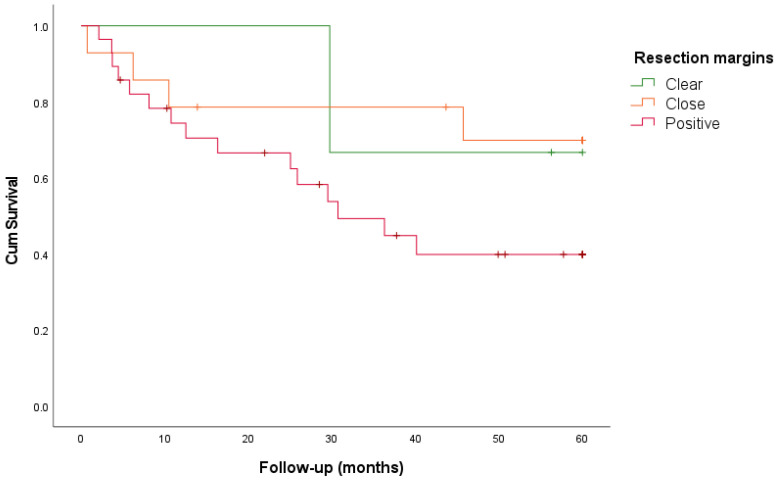
Relationship between disease-specific survival and resection margin in primary hypopharyngeal squamous cell carcinoma.

In total 268 patients were included in the study: 107 with primary LSCC, 100 with recurrent LSCC, 45 with primary HSCC, and 16 with recurrent HSCC. The clinico-pathological characteristics are shown in Table 1. The resection margins of these four different tumor groups were analyzed according to the RCP guidelines. Clear, close, and positive resection margins were identified for each resection surface (cranial, ventral, lateral, dorsal, and caudal), and are reported in Table 2. The results regarding recurrence and survival are summarized in Table 3 and Figure 1, Figure 2 and Figure 3.

### 3.2. Results Primary LSCC

One hundred and seven patients underwent TL or TLP. The resection margins were clear (>5 mm) in 19%, close (1–5 mm) in 27%, and positive (<1 mm) in 54% of cases, with TRs of 5%, 24%, and 36%, respectively. A linear association was observed between TR and narrowing margins (linear-by-linear association, *p* = 0.007). LR was found in 5% of clear, 0% of close, and 16% of positive margins. There was no increase in the LR rate for narrowing margins (linear-by-linear association, *p* = 0.058). The 5-year DSS rates for clear, close, and positive margins were 95%, 78%, and 63%, respectively (log-rank test, *p* = 0.041). The 5-year DFS rates for clear, close, and positive margins were 55%, 45%, and 39%, respectively (*p* = 0.776).

### 3.3. Results Recurrent LSCC

One hundred patients underwent TL or TLP. The resection margins were clear in 37%, close in 34%, and positive in 29% of the cases, with TRs of 35%, 38%, and 69%, respectively. A linear association was observed between TR and narrowing margins (*p* = 0.008). LR was found in 14% of clear, 21% of close, and 45% of positive margins. There was an increased LR rate for narrowing margins (*p* = 0.004). The 5-year DSS rates for clear, close, and positive margins were 61%, 64%, and 26%, respectively (*p* = 0.002). The 5-year DFS rates for clear, close, and positive margins were 40%, 43%, and 10%, respectively (*p* = 0.002).

A multivariate survival analysis for primary and recurrent laryngeal tumors (n = 207) with confounders including age, primary, or recurrent tumor, pT1 and 2 or pT3 and 4, pN0 or pN+, and postoperative adjuvant treatment showed a worse DFS (hazard ratio (HR) 1.7, 95% CI 1.1 to 2.8, *p* = 0.020) and DSS (HR 1.7, 95% CI 1.0 to 2.7, *p* = 0.041) in cases with positive margins compared to those with clear margins. There were no differences between close and clear margins (DFS HR 0.9, 95% CI 0.6 to 1.5; DSS HR 0.9, 95% CI 0.5 to 1.4).

### 3.4. Results Primary HSCC

Forty-five patients underwent TL or TLP. The resection margins were clear in 7%, close in 31%, and positive in 62% of the cases, with TRs of 33%, 29%, and 54%, respectively. There was no linear association between TR and narrowing margins (*p* = 0.165). LR was found in 33% of clear, 7% of close, and 18% of positive margins. There was no increase in the LR rate in the case of narrowing margins (*p* = 0.942). The 5-year DSS rates for clear, close and positive margins were 67%, 70%, and 40%, respectively (*p* = 0.207). The 5-year DFS rates for clear, close, and positive margins were 33%, 50%, and 18%, respectively (*p* = 0.030).

### 3.5. Results Recurrent HSCC

Sixteen patients underwent TL or TLP. The resection margins were clear in 25%, close in 31%, and positive in 44% of the cases, with TRs of 50%, 60%, and 71%, respectively. There was no linear association between TR and narrowing margins (*p* = 0.486). LR was found in 25% of clear, 60% of close, and 43% of positive margins, resulting in no difference in LR rates (*p* = 0.678). Survival analysis was not performed because the number of patients was too small.

Multivariate survival analysis in both HSCC groups (n = 61) with confounders, age, primary or recurrent tumor, pT1 and 2 or pT3 and 4, pN0 or pN+, and postoperative adjuvant treatment, did not show an association between resection margin status and survival (for positive vs. clear margins, DFS HR 2.3, 95% CI 0.7 to 7.0, and DSS HR 2.4, 95% CI 0.8 to 7.4).

## 4. Discussion

The larynx and hypopharynx have a complex anatomy and the achievement of a >5 mm resection margin is limited. In this study, the percentage of positive resection margins was remarkably high for both primary and recurrent LSCC and HSCC (54% for primary LSCC, 29% for recurrent LSCC, 62% for primary HSCC, and 44% for recurrent HSCC). Here, we discuss the resection margins of different resection surfaces and their anatomical limitations. Resection margins in relation to the cranial and caudal surfaces were analyzed for LSCC and HSCC without distinguishing between primary and recurrent tumors. Caudal resection surfaces showed low numbers of positive margins for LSCC (2%), HSCC (3%), and close margins (LSCC 4% and HSCC 7%) (Table 2). At the caudal site (trachea or esophagus), resection margins of >5 mm for both LSCC and HSCC are always feasible because of the anatomy. The percentages of positive margins for cranial resection surfaces in this cohort were higher: 8% for LSCC and 11% for HSCC. In addition, 12% had close margins for both LSCC and HSCC. However, at the cranial site, a margin of >5 mm should always be feasible because, at that location, an additional mucosal resection could be performed when needed. Resection margins in relation to the other surfaces (ventral, dorsal, and lateral) were analyzed separately for LSCC and HSCC owing to their delicate anatomy (Table 2). For the ventral resection surface, a positive margin of 31% and a close margin of 29% were found in LSCC. In cases of extra-laryngeal tumor growth and/or cartilage invasion, ventral margins of >5 mm cannot be achieved because the strap muscles are <5 mm thick. Skin resection should only be performed in cases of skin involvement because of the morbidity associated with reconstruction. Therefore, a close ventral resection margin should be accepted in patients without skin involvement. For HSCC, only piriform sinus and postcricoid tumors with endolaryngeal invasion have a ventral resection surface. The ventral resection margin was positive in 34% of cases and close in 28% of cases. For the dorsal resection surface, the positive and close margins were both 7% in LSCC. The dorsal resection surface in LSCC is confined only by the postcricoid mucosa because the adjacent lumen of the hypopharynx and esophagus are not resection surfaces. Achieving a resection margin > 5 mm in this area is not feasible because the thickness of the dorsal laryngeal tissue (mucosa and submucosa) is only 2 mm. For HSCC, a positive dorsal resection margin of 12% and a close resection margin of 9% were observed. The dorsal resection margin for HSCC only exists for anterior and lateral wall piriformis sinus tumors or posterior pharyngeal wall tumors. However, only the thickness of the tissue (mucosa and submucosa) of the anterior wall of the piriform sinus allows for a resection margin of >5 mm. Tumors at the medial wall of the piriform sinus and postcricoid do not have a dorsal resection surface because of the adjacent lumen of the hypopharynx and esophagus. For LSCC, a positive lateral resection surface was found in 2% of the cases and close in 7% of the cases. Lateral margins of >5 mm should always be feasible because of the piriform sinus anatomy. For HSCC, a positive lateral resection surface was found in 3% of the cases and close in 12% of the cases (Table 2). For lateral and anterior piriform sinus tumors, this resection surface is relevant, but a margin of >5 mm is not feasible because of the limited thickness of the mucosa and submucosa and its direct relationship with vital vascular structures. A resection margin of >5 mm is feasible only for tumors of the posterior pharyngeal wall and the postcricoid.

Furthermore, we assessed the clinical prognostic relevance of resection margins by analyzing the recurrence and survival rates. Given the different prognoses of LSCC and HSCC and the different anatomical characteristics, primary and recurrent LSCC and HSCC were analyzed separately in the four groups. For LSCC, we found a significant association between TR and narrowing margin for primary (*p* = 0.007) and recurrent tumors (*p* = 0.008). In addition, the narrowing of the margin was associated with an increased LR rate for recurrent LSCC (*p* = 0.004). In contrast, for both primary and recurrent HSCC, a narrowing margin showed no association with TR or LR. It can be argued that resection margins mostly influence LR and not regional recurrence or distant metastasis. Basheeth et al. found a significant association (univariate analysis, *p* < 0.001) between positive margins and LR in the neopharynx (base of the tongue/pharynx) compared to LR around the stoma (*p* = 0.45) for primary and recurrent LSCC [10]. In this study, we only found a higher LR rate (mucosal neopharynx or stomal recurrences) for positive margins in patients with recurrent LSCC (*p* = 0.004), but not for primary LSCC. This is probably because patients with primary LSCC received postoperative therapy after TL or TLP. Adjuvant therapy was mainly postoperative RT and in some cases CRT, RT with hyperthermy or proton therapy. Positive margins were associated with a significantly worse 5-year DSS for primary (*p* = 0.041), and recurrent LSCC (*p* = 0.001). This was also the case for the 5-year DFS in patients with recurrent LSCC (*p* = 0.002) and primary HSCC (*p* = 0.030). In contrast to positive resection margins, the survival rates for close and clear margins were comparable between primary and recurrent LSCC (DSS) and primary HSCC (DFS), which could imply that close and clear margins are similar in terms of survival. This may suggest that a clear margin is not always feasible because of the anatomy of the larynx and hypopharynx, and is not clinically relevant in terms of survival. Multivariate survival analysis for LSCC (primary and recurrent) showed that positive margins were independent negative predictors of DFS and DSS. Histological tumor characteristics (e.g., differentiation grade, perineural growth, and angio-invasion) were not consistently reported and were therefore not included in this analysis. Next to this, the variability in TNM stage, tumor location, and pre- and postsurgical treatment in this retrospective study may also influence the prognosis. For HSCC, positive margin status was not found to be an independent prognostic factor. However, firm conclusions could not be drawn because of the small number of patients.

Unfortunately, in retrospective studies, it is not possible to determine whether pathological assessment was performed consistently and according to the RCP. Next, the resection margins for each resection surface were not always available in the pathology reports (particularly the dorsal and lateral resection margins, which were often unknown). The RCP guidelines for the larynx describe how to record a histopathology report, whereby the resection margins are defined as clear > 5 mm, close 1–5 mm, and positive < 1 mm. How the pathological examination should be performed, such as the required orientation of the different resection surfaces or how to measure these margins, is not mentioned. The importance of anatomical orientation of the resection specimen, accurate identification of different resection surfaces, and measurement of resection margins is crucial [9]. A standardized pathological assessment and report is needed before a definite statement on patient prognosis can be made for LSCC and HSCC. Clear communication and collaboration between the pathologist and surgeon are key.

A literature search revealed a lack of studies regarding the clinical relevance of resection margins in laryngeal and hypopharyngeal oncologic surgery. Only 12 studies on resection margins during TL/TLP have been found. Only one study has followed the RCP resection margin guidelines for recurrent LSCC and HSCC [8]. The authors reported 72% clear, 18% close, and 9% positive margins, and a 5-year DFS of 55% and DSS of 55% for the total population, regardless of the margin status. Compared to our study of patients with recurrent LSCC and HSCC, the percentage of close and positive margins was higher (35% clear, 34% close, and 31% positive margins), and the 5-year DFS (31%) and DSS (49%) were lower. The DFS in another study (not according to the RCP guidelines) was 63% for primary and 47% for recurrent LSCC and HSCC [11]. The DSS rates in other studies (not according to the RCP guidelines) were 58% (primary and recurrent LSCC) [10], 80% (recurrent LSCC) [12], 52% (primary HSCC) [13], 46% (primary HSCC; TL or RT as treatment) [14], 63% (primary LSCC; TL) [15], and 58% (primary LSCC; TL and RT) [15]. Two studies defined close margins as <5 mm and positive margins as tumor at the resection surface [13,15]. These studies reported 77–81% clear, 9–14% close, and 5–11% positive margins. LR was reported in 18–24% and 5-year DSS in 52–63%, regardless of margin status. The remaining nine articles used descriptive definitions for margin status, such as ‘positive’, ‘microscopically positive’, ‘tumor at the resection surface’, ‘negative’, ‘safe margins’ or ‘no invasive tumor at the resection surface’. These studies reported clear margins in 70–100% and positive margins in 0–30% [10,11,12,14,16,17,18,19,20]. The results of our survival data cannot be compared with those in the literature because diverse patient cohorts and descriptive definitions for margin status have been used in different studies. The patient cohorts varied in tumor location, TNM classification, and pre- and postoperative treatment. Saraniti et al. confirmed that a comparison of the literature is not possible, and to investigate the prognostic value of resection margins, a meta-analysis should be performed with identical definitions of resection margins, methodology, and postoperative treatment [6]. This study is unique because we analyzed separate groups (primary/recurrent and LSCC/HSCC) and used the RCP guidelines. Despite the lack of a meta-analysis, it is needless to say that margins of <1 mm should be strictly avoided, while a margin of 1–5 mm could be accepted in specific cases, as shown in this study.

## 5. Conclusions

To the best of our knowledge, this study is the first to analyze the relationship between resection margins of different resection surfaces and the prognostic value in distinctive groups: primary and recurrent LSCC and HSCC. For primary and recurrent LSCC, significantly more (local) recurrences were found in cases with narrowing margins, and positive margins were an independent predictor of worse DFS and DSS in a multivariate survival analysis. The survival rates for close and clear margins were comparable for primary and recurrent LSCC implying that close and clear margins are similar in terms of survival. This may suggest that a margin > 5 mm is not clinically relevant in terms of survival. Therefore, a margin of 1–5 mm should be accepted for certain subsites. Margins < 1 mm should be avoided, particularly in salvage surgery. Histopathological assessment of laryngeal and/or hypopharyngeal resection specimens should be universal to draw definitive conclusions about the influence of resection margins on patient outcomes.

## Figures and Tables

**Table 1:** Patient and tumor characteristics.

Characteristics	Primary LSCC N = 107	Recurrent LSCC N = 100	Primary HSCC N = 45	Recurrent HSCC N = 16	*p*-Value *
**Age (years), mean (SD)**	65 (10)	65 (11)	65 (9)	62 (7)	0.67
**Sex**					0.86
Male	87 (81)	80 (80)	35 (78)	14 (88)	
Female	20 (19)	20 (20)	10 (22)	2 (12)	
**Tumor location**					0.29
Supraglottic	50 (47)	36 (36)			
Glottic	53 (50)	59 (59)			
Subglottic	4 (3)	5 (5)			
Piriform sinus			30 (67)	11 (69)	0.95
Posterior pharyngeal wall			4 (9)	1 (6)	
Postcricoid			11 (24)	4 (25)	
**pT classification**					<0.001
pT1 and 2	5 (5)	32 (32)	5 (11)	7 (44)	
pT3 and 4	102 (95)	68 (68)	40 (89)	9 (56)	
**pN classification**					0.002
pN0	68 (64)	81 (81)	25 (56)	14 (87)	
pN+ (pN1 and 2)	39 (36)	19 (19)	20 (44)	2 (13)	
**Type of surgery**					<0.001
TL	99 (93)	93 (93)	9 (20)	2 (13)	
TLP	8 (7)	7 (7)	36 (80)	14 (87)	
**Perineural invasion**					0.20
No	65 (66)	51 (53)	24 (53)	7 (47)	
Yes	34 (34)	46 (47)	21 (47)	8 (53)	
Unknown	8	3	-	1	
**Angio-invasion**					0.55
No	59 (60)	60 (66)	23 (54)	9 (56)	
Yes	39 (40)	31 (34)	20 (46)	7 (44)	
Unknown	9	9	2	-	
**Infiltrative growth pattern**					0.73
No	13 (19)	11 (14)	5 (14)	1 (8)	
Yes	55 (81)	66 (86)	31 (86)	11 (92)	
Unknown	39	23	9	4	
**Cartilage invasion**					0.001
No	28 (26)	44 (44)	13 (29)	11 (69)	
Yes	79 (74)	56 (56)	32 (71)	5 (31)	
**Preoperative therapy**					**
RT	-	77 (77)	-	5 (31)	
CRT	-	18 (18)	-	10 (63)	
TLM	-	3 (3)	-	-	
TLM + RT	-	2 (2)	-	1 (6)	
**Postoperative therapy**					<0.001
None	16 (15)	96 (96)	16 (36)	16 (100)	
Yes ***	91 (85)	4 (4)	29 (64)	0 (0)	

LSCC: laryngeal squamous cell carcinoma, HSCC: hypopharyngeal squamous cell carcinoma, SD: standard deviation, TL: total laryngectomy, TLP: total laryngopharyngectomy, RT: radiotherapy, CRT: chemoradiotherapy, TLM: transoral laser microsurgery. For categorical variables numbers and percentages of valid cases in each category are presented. * *p*-value for difference between four or two groups. ** Too small amount. *** RT, CRT, RT, and hyperthermy or proton therapy.

**Table 2:** Percentage in resection margin status for each resection surface.

		All Resection Surfaces	Cranial	Ventral	Lateral	Dorsal	Caudal
**Laryngeal SCC** (n = 207)	Clear	28%	53%	32%	25%	10%	94%
	Close	30%	12%	29%	7%	7%	4%
	Positive	42%	8%	31%	2%	7%	2%
	Unknown	-	27%	7%	66%	76%	0%
**Hypopharyngeal SCC** (n = 61)	Clear	12%	52%	28%	22%	0%	90%
	Close	31%	12%	28%	12%	9%	7%
	Positive	57%	11%	34%	3%	12%	3%
	Unknown	-	24%	10%	68%	75%	0%

**Table 3:** Total and local recurrence for resection margins according to the RCP guidelines.

	Margins	Total	Recurrence	Local Recurrence
**Primary LSCC** (n = 107)	Clear	19% (20)	5% (1)	5% (1)
	Close	27% (29)	24% (7)	0% (0)
	Positive	54% (58)	36% (21)	16% (9)
**Recurrent LSCC** (n = 100)	Clear	37% (37)	35% (13)	14% (5)
	Close	34% (34)	38% (13)	21% (7)
	Positive	29% (29)	69% (20)	45% (13)
**Primary HSCC** (n = 45)	Clear	7% (3)	33% (1)	33% (1)
	Close	31% (14)	29% (4)	7% (1)
	Positive	62% (28)	54% (15)	18% (5)
**Recurrent HSCC** (n = 16)	Clear	25% (4)	50% (2)	25% (1)
	Close	31% (5)	60% (3)	60% (3)
	Positive	44% (7)	71% (5)	43% (3)

RCP: Royal College of Pathologists, LSCC: laryngeal squamous cell carcinoma, HSCC: hypopharyngeal squamous cell carcinoma.

## Data Availability

The data presented in this study is available on request from the corresponding author due to privacy reasons.

## References

[1] Hamoir M., Holvoet E., Ambroise J., Lengelé B., Schmitz S. (2017). Salvage surgery in recurrent head and neck squamous cell carcinoma: Oncologic outcome and predictors of disease free survival. Oral Oncol..

[2] Elbers J.B.W., Veldhuis L.I., Bhairosing P.A., Smeele L.E., Jóźwiak K., van den Brekel M.W.M., Verheij M., Al-Mamgani A., Zuur C.L. (2019). Salvage surgery for advanced stage head and neck squamous cell carcinoma following radiotherapy or chemoradiation. Eur. Arch. Otorhinolaryngol..

[3] Helliwell T., Woolgar J., The Royal College of Pathology Standards and Datasets for Reporting Cancers. Dataset for Histopathology Reporting of Mucosal Malignancies of the Larynx. November 2013. https://www.rcpath.org/static/0d6c0512-e285-40fd-b8a9ee31b13887de/Dataset-for-histopathology-reporting-of-mucosal-malignancies-of-the-larynx.pdf.

[4] Bradford C.R., Wolf G.T., Fisher S.G., McClatchey K.D. (1996). Prognostic importance of surgical margins in advanced laryngeal squamous carcinoma. Head Neck..

[5] Fowler B.Z., Muller S., Chen A.Y., Johnstone P.A. (2006). Factors influencing long-term survival following salvage total laryngectomy after initial radiotherapy or conservative surgery. Head Neck..

[6] Saraniti C., Speciale R., Gallina S., Salvago P. (2019). Prognostic role of resection margin in open oncologic laryngeal surgery: Survival analysis of a cohort of 139 patients affected by squamous cell carcinoma. Braz. J. Otorhinolaryngol..

[7] Gallo A., Manciocco V., Simonelli M., Pagliuca G., D’Arcangelo E., de Vincentiis M. (2005). Supracricoid partial laryngectomy in the treatment of laryngeal cancer: Univariate and multivariate analysis of prognostic factors. Arch. Otolaryngol. Head Neck Surg..

[8] Wulff N.B., Andersen E., Kristensen C.A., Sørensen C.H., Charabi B., Homøe P. (2017). Prognostic factors for survival after salvage total laryngectomy following radiotherapy or chemoradiation failure: A 10-year retrospective longitudinal study in eastern Denmark. Clin. Otolaryngol..

[9] Bernard S.E., van Lanschot C.G.F., Hardillo J.A., Monserez D.A., Meeuwis C.A., Baatenburg de Jong R.J., Koljenović S., Sewnaik A. (2024). A new proposal for adequate resection margins in larynx and hypopharynx tumor surgery. Cancers.

[10] Basheeth N., O’Leary G., Khan H., Sheahan P. (2015). Oncologic outcomes of total laryngectomy: Impact of margins and preoperative tracheostomy. Head Neck..

[11] Tassone P., Savard C., Topf M.C., Keane W., Luginbuhl A., Curry J., Cognetti D. (2018). Association of Positive Initial Margins With Survival Among Patients With Squamous Cell Carcinoma Treated With Total Laryngectomy. JAMA Otolaryngol. Head Neck Surg..

[12] de Vincentiis M., De Virgilio A., Bussu F., Gallus R., Gallo A., Bastanza G., Parrilla C., Greco A., Galli J., Turchetta R. (2015). Oncologic results of the surgical salvage of recurrent laryngeal squamous cell carcinoma in a multicentric retrospective series: Emerging role of supracricoid partial laryngectomy. Head Neck..

[13] Bova R., Goh R., Poulson M., Coman W.B. (2005). Total pharyngolaryngectomy for squamous cell carcinoma of the hypopharynx: A review. Laryngoscope.

[14] Mochiki M., Sugasawa M., Nibu K., Asai M., Nakao K., Asakage T. (2007). Prognostic factors for hypopharyngeal cancer: A univariate and multivariate study of 142 cases. Acta Otolaryngol. Suppl..

[15] Spector J.G., Sessions D.G., Lenox J., Simpson J. (2006). Management of T3N1 glottic carcinoma: Therapeutic outcomes. Laryngoscope.

[16] Zhao H., Ren J., Zhuo X., Ye H., Zou J., Liu S. (2009). Stomal recurrence after total laryngectomy: A clinicopathological multivariate analysis. Am. J. Clin. Oncol..

[17] Zhang X., Liu Z., Li Q., Liu X., Li H., Liu W., Li Q., Guo Z., Zeng Z. (2013). Using a linear stapler for pharyngeal closure in total laryngectomy. Eur. Arch. Otorhinolaryngol..

[18] Antin F., Breheret R., Goineau A., Capitain O., Laccourreye L. (2021). Rehabilitation following total laryngectomy: Oncologic, functional, socio-occupational and psychological aspects. Eur. Ann. Otorhinolaryngol. Head Neck Dis..

[19] Tsai M.H., Chuang H.C., Lin Y.T., Huang T.L., Fang F.M., Lu H., Chien C.Y. (2021). Survival Outcomes and Predictors for Patients who Failed Chemoradiotherapy/Radiotherapy and Underwent Salvage Total Laryngectomy. Int. J. Environ. Res. Public Health.

[20] Mucha-Małecka A., Biesaga B., Janecka-Widła A., Przewoźnik M., Małecki K. (2021). Assessment of HPV16 infection in patients with laryngeal cancer. Pol. J. Pathol..

